# Compensatory Mutations in Predicted Metal Transporters Modulate Auxin Conjugate Responsiveness in *Arabidopsis*

**DOI:** 10.1534/g3.112.004655

**Published:** 2013-01-01

**Authors:** Rebekah A. Rampey, Megan T. Baldridge, David C. Farrow, Sarah N. Bay, Bonnie Bartel

**Affiliations:** * Department of Biology, Harding University, Searcy, Arkansas 72149; † Department of Biochemistry and Cell Biology, Rice University, Houston, Texas 77005

**Keywords:** MTP5, IAR1, auxin conjugates, metal transport, Arabidopsis

## Abstract

Levels of the phytohormone indole-3-acetic acid (IAA) can be altered by the formation and hydrolysis of IAA conjugates. The isolation and characterization of *Arabidopsis thaliana* mutants with reduced IAA-conjugate sensitivity and wild-type IAA responses is advancing the understanding of auxin homeostasis by uncovering the factors needed for conjugate metabolism. For example, the discovery that the IAA-Ala-resistant mutant *iar1* is defective in a protein in the ZIP family of metal transporters uncovered a link between metal homeostasis and IAA-conjugate sensitivity. To uncover additional factors impacting auxin conjugate metabolism, we conducted a genetic modifier screen and isolated extragenic mutations that restored IAA-amino acid conjugate sensitivity to the *iar1* mutant. One of these suppressor mutants is defective in a putative cation diffusion facilitator, MTP5 (At3g12100; formerly known as MTPc2). Loss of MTP5 function restored IAA conjugate sensitivity to *iar1* but not to mutants defective in IAA-amino acid conjugate amidohydrolases. Our results are consistent with a model in which MTP5 and IAR1 transport metals in an antagonistic fashion to regulate metal homeostasis within the subcellular compartment in which the IAA-conjugate amidohydrolases reside, and support previous suggestions that the ion composition in this compartment influences hydrolase activity.

Certain trace elements are essential for plant growth and development. For example, Cu^2+^ and Mn^2+^ are used in many electron transfer reactions, including those in photosynthesis, Zn^2+^ ions provide structure to DNA-binding proteins and serve as cofactors for hydrolytic enzymes, and iron is essential for heme proteins such as ferredoxin and catalase [reviewed in (Clemens *et al.* 2002; Hall and Williams 2003)]. When appropriate levels of any of these ions are not maintained, plants display an array of symptoms, including reduced growth (Marschner 1995).

Numerous cation transporters facilitate ion homeostasis. In *Arabidopsis*, there are at least 880 putative membrane transporters that fall into 46 families (Mäser *et al.* 2001). Disruption of some of these transporters leads to measurable metal-related phenotypes in single mutants (Cheng *et al.* 2003; Delhaize *et al.* 2007; Li *et al.* 2008; Kawachi *et al.* 2009). However, not all mutants defective in putative metal transporters display morphological or ionomic phenotypes due to the complexity and redundancy of the elaborate metal transport system. Establishing function for some of these predicted transporters may require alternative genetic, biochemical, or physiological analyses.

Auxin is a plant hormone that controls a plethora of growth and developmental processes, and auxin metabolism is complex and distributed among several subcellular compartments (Woodward and Bartel 2005; Strader and Bartel 2011). By screening for *Arabidopsis* mutants with defective indole-3-acetic acid (IAA) conjugate responsiveness, we uncovered several enzymes that hydrolyze conjugates (Bartel and Fink 1995; Davies *et al.* 1999; Rampey *et al.* 2004). These IAA-amino acid hydrolases contain predicted N-terminal signal sequences and C-terminal endoplasmic reticulum (ER) retention signals (Bartel and Fink 1995; Davies *et al.* 1999), and several localize to the ER in organelle proteomics experiments (Dunkley *et al.* 2006). The observation that the IAA-amino acid hydrolases are metalloenzymes (Bartel and Fink 1995; Davies *et al.* 1999; Rampey *et al.* 2004; Bitto *et al.* 2009) may explain why proteins that are likely to influence metal transport also have emerged from screens for reduced IAA-conjugate responsiveness. Analyses of the IAA-conjugate response mutants *iaa-leucine resistant2* (*ilr2*) (Magidin *et al.* 2003), *ilr3* (Rampey *et al.* 2006), and *iaa-alanine resistant1* (*iar1*) (Lasswell *et al.* 2000) reveal a critical role for the metal microenvironment in IAA-conjugate metabolism and indicate that an understanding of subcellular metal homeostasis will be required to fully elucidate mechanisms regulating IAA levels. For example, *ilr2* seedlings are resistant to root growth inhibition not only by IAA conjugates but also by Mn^2+^ and Co^2+^ (Magidin *et al.* 2003). ILR2 (At3g18485) is an apparently cytosolic protein that may inhibit an unidentified metal transporter, as *ilr2* microsomes have enhanced Mn^2+^ transport activity compared with wild-type microsomes (Magidin *et al.* 2003). In addition, the ILR3 (At5g54680) basic helix–loop–helix leucine zipper (bHLH105) transcription factor regulates transcription of metal transporter genes (Rampey *et al.* 2006) that appear to modulate iron distribution (Gollhofer *et al.* 2011). A dominant gain-of-function *ilr3* allele reduces IAA-Leu and Mn^2+^ responsiveness, whereas loss of *ILR3* heightens responses to both IAA-Leu and Mn^2+^ (Rampey *et al.* 2006).

The *iar1* mutant has decreased sensitivity to a variety of IAA conjugates (Lasswell *et al.* 2000) that are *in vitro* substrates of the *Arabidopsis* IAA-amino acid hydrolases (LeClere *et al.* 2002). IAR1 (At1g68100) contains at least seven predicted transmembrane domains and many His-rich regions, consistent with a role in metal binding or transport (Lasswell *et al.* 2000). IAR1 resembles members of the zinc-regulated transporter iron-regulated transporter-like protein (ZIP) family that transport metals from the vacuole or apoplast into the cytosol (Gaither and Eide 2000). Although there are at least 15 ZIP family members in *Arabidopsis* (Hall and Williams 2003), IAR1 is the only *Arabidopsis* member of the LIV-1 zinc transporter (LZT) subfamily (Taylor and Nicholson 2003). Heterologous expression of mouse LZT protein ZIP7/KE4, which is 26% identical to IAR1, complements the *Arabidopsis iar1* mutant (Lasswell *et al.* 2000) and the *Saccharomyces cerevisiae yke4* mutant (Kumánovics *et al.* 2006), suggesting that *IAR1*, *ZIP7*, and *YKE4* are orthologs that function similarly in plants, animals, and yeast. Although the localization and substrate specificity of *Arabidopsis* IAR1 have not been determined, mammalian ZIP7/KE4 is a zinc exporter localized in the Golgi membrane (Huang *et al.* 2005), and yeast Yke4p is a bidirectional zinc transporter localized in the ER membrane (Kumánovics *et al.* 2006), consistent with the possibility that IAR1 is a zinc transporter in the secretory pathway. Determining the function of IAR1 may provide insight into IAA-conjugate responses and metabolism in plants and contribute to understanding the function of other members of the LZT transporter family.

The observation the IAA-conjugate hydrolases require metal cofactors for activity (Bartel and Fink 1995; Davies *et al.* 1999; Rampey *et al.* 2004) underlies the hypothesis that the direct consequences of *ilr2*, *ilr3*, and *iar1* mutations are alterations in ion homeostasis, and the altered sensitivity to IAA conjugates in these mutants is a secondary effect of this change. Indeed, exogenous manganese suppresses the IAA-Ala resistance of *iar1* (Lasswell *et al.* 2000), and ion level alterations are detected in *ilr3* mutants grown on certain media (Rampey *et al.* 2006). However, ionomic changes have not been identified in either *ilr2* (Magidin *et al.* 2003) or *iar1* (Lasswell *et al.* 2000) plants, and *iar1* mutants lack morphological phenotypes expected from a dramatic ion imbalance, suggesting that any such ionomic changes are modest or restricted to specific tissues or subcellular locations.

We have taken a genetic approach to clarify the role of IAR1 in IAA-conjugate sensitivity. We conducted an *iar1* suppressor screen to isolate genes that when defective restore wild-type IAA-conjugate sensitivity to *iar1* roots. We identified *mtp5* as a mutant defective in a cation diffusion facilitator family transporter that suppressed the IAA-conjugate resistance of *iar1* but not other IAA-conjugate sensitivity mutants. The isolation of *mtp5* as an *iar1* suppressor supports the hypothesis that IAR1 functions as a metal transporter and suggests that MTP5 and IAR1 transport ions in an antagonistic fashion to control subcellular metal homeostasis.

## Materials and Methods

### Plant materials and growth conditions

Plants from the Columbia (Col-0) and Wassilewskija (Ws) accessions were used. For phenotypic assays, seeds were surface-sterilized (Last and Fink 1988) and grown aseptically on plant nutrient medium containing 0.5% (w/v) sucrose [PNS (Haughn and Somerville 1986)] solidified with 0.6% agar. Seedlings were grown in medium alone or medium supplemented with IAA, IAA-l-amino acid conjugates (Sigma-Aldrich) or other hormones (from 0.1, 1, or 100 mM stocks in ethanol), or Basta [glufosinate ammonium, Crescent Chemical, Augsburg, Germany; from a 50 mg/mL stock in 25% (v/v) ethanol]. Media supplemented with metals (from 100 mM MnCl_2_, 500 mM CoCl_2_, 2 M CaCl_2_, 20 mM CdCl_2_, and 500 mM ZnSO_4_ stocks in H_2_O) were prepared without sucrose. Plates were sealed with gas-permeable Leukopor surgical tape (LecTec Corp, Minnetonka, MN) and incubated with constant illumination (25 to 45 µEm^-2^s^-2^) at 22° under yellow long-pass filters to slow the breakdown of indolic compounds (Stasinopoulos and Hangarter 1990). Plants transferred to soil (Metromix 200, Scotts, Marysville, OH) were grown at 22 to 25° under continuous illumination by Cool White fluorescent bulbs (Sylvania, Danvers, MA).

### Mutant isolation

Approximately 48,000 progeny of *iar1-3* (Col-0 accession) mutagenized with ethyl methanesulfonate (Normanly *et al.* 1997) and *iar1-1* (Ws accession) mutagenized with fast-neutron bombardment (Lasswell 2000) were screened for *iar1* suppressors by plating ~1000 seeds per 150 mm plate on PNS supplemented with 40 μM IAA-Ala. Seedlings were screened after growing for 8 d in continuous yellow-filtered light at 22°. Seedlings with wild-type sensitivity to IAA-Ala were transferred to PNS and allowed to recover, and seedlings with roots that failed to elongate after transfer were discarded. The remaining mutants were transferred to soil for seed production. Progeny of these plants were tested on 40 μM IAA-Ala and PNS. Homozygous *iar1* mutations in the suppressors were confirmed using polymerase chain reaction (PCR) amplification and restriction analyses. The *iar1-1* mutation was detected as described previously (Lasswell *et al.* 2000), and the *iar1-3* mutation was detected by PCR amplification with the oligonucleotides 5′-GAACCAGGACAATCATCGTTG-3′ and 5′-CCCAAGCTTGGGATTTCTATATCGGTTAC-3′ followed by digestion of the resulting product with *Hae*III to yield a 328-bp product for Col-0 and 258-bp and 70-bp products for *iar1-3*.

The *mtp5-1* mutant was isolated as suppressor of *iar1-3*, a loss-of-function *iar1* allele in the Col-0 accession (Lasswell *et al.* 2000). To maintain *iar1* in the mapping population, the N13 (*mtp5-1 iar1-3*) isolate was outcrossed to *iar1-1* [in the Ws accession (Lasswell *et al.* 2000)]. The F_2_ progeny from this outcross were plated on 40 μM IAA-Ala, and seedlings displaying wild-type root lengths were selected for PCR-based recombination mapping. The causative mutation was located to the top of chromosome 3 using the published markers nga172 and nga162 (Bell and Ecker 1994). To further delineate the mapping interval, new PCR-based markers were designed (Table 1).

**Table 1:** New markers used in the positional cloning of *MTP5*

Marker*^a^*	Oligonucleotides (5′ to 3′)	Enzyme	Size of Product, bp
F13M14-3+4	F13M14-3*^b^* CTTCTTCTATATTGAGTAGGTAGATTAAAA	*Bsa*BI	Col – 226
	F13M14-4 ATTACCTAAAGACTCTGATTTTATACTCTC		Ws – 196, 30
T7M13-3+4	T7M13-3 AGTTAACCAACGATAACAAGCAGATTCGTT	*Bcl*I	Col – 130, 20
	T7M13-4 CTCCTACAATCCTCTAAGAATCCATTGATC		Ws – 150
F24K9-7+8	F24K9-7 AATTTAAAATTATATGCAAACTAATTAGAT	*Bgl*II	Col – 200 + 30
	F24K9-8 GTAGCTAAAAAGTTGCTGCAAGCAAGGAAA		Ws – 230
F26K24-9+6	F26K24-6 GATAATAACGAAGAGTATGAAGTAAAAGTA	*Hpy*CHIV	Col – 523
	F26K24-9 AGATTCGATTACACTAGGCAATTTGTTATGA		Ws – 128, 395
MEC18-7+8	MEC18-7 TTCGATTCAAGACAAAGTTTAAAGTTACAA	*Bsp*HI	Col – 220
	MEC18-8*^b^* AATACTTTAAGTTTTGGATGTAAGATTCAT		Ws – 190, 30
F28J15-3+4	F28J15-3 AATATCGGCCAACAGTAAGTT	*Hin*P1I	Col – 400, 200
	F28J15-4 CATCACGTAACTGAGATTCC		Ws – 600
T2E22-9+10	T2E22-9*^b^* AGCCCTATGCACACACATGTAAAAATGGGA	*Fok*I	Col – 213
	T2E22-10 ATTCACTGATTTATTTGTTACCTAGCTAAA		Ws – 172, 41
MBK21-10+11	MBK21-10 GATACTCAAGTAGTTATCTGTTACCTTTAG	*Hin*cII	Col – 350, 150
	MBK21-11 CTAAACCATTATGTGTAATGTGTGAATTAG		Ws – 500
MGH6-2+3	MGH6-2 TAGTTTCTCTGATTACTTGTGTAGATGTGA	*Mbo*II	Col – 357
	MGH6-3*^b^* TATAGCTGTTCCAAGACTATAACACCGGAA		Ws – 327, 30
MRP15-3+4	MRP15-3 GTTAGAATTGGAATTAACAAGTATTACTAG	*Taq*I	Col – 251, 56
	MRP15-4 CTTGATAGCATTGGGAGCAAGCAACGAACC		Ws – 213, 56, 41

Col-0, Columbia; dCAPS, derived cleaved amplified polymorphic sequences; PCR, polymerase chain reaction; Ws, Wassilewskija.

a Markers reveal polymorphisms between Col-0 and Ws accessions when cut with the indicated restriction enzymes after PCR amplification with the indicated oligonucleotides.

b This is a dCAPS oligonucleotide (Michaels and Amasino 1998; Neff *et al.* 1998); the underlined nucleotide differs from wild-type sequence to create a restriction site in either the Col-0 or Ws PCR product.

A candidate gene within the mapping region, *At3g12100*, was PCR-amplified and sequenced using DNA from an N13 mapping plant with the following oligonucleotides: T21B14-7 (5′-ATAAAGAATACAACTTTTTCTAGCTTTTAG-3′) and T21B14-9 (5′-TGAATAAAAGTGTCTTCTTGCTTGACTACA-3′), T21B14-13 (5′-GGAAATATGTACACATTCGAGGAACGATT-3′) and T21B14-17 (5′-CTTTGATTTGTTTAATATTTGACATATGTG-3′), T21B14-18 (5′-TATGAGCATCAATTCATACAAGTCTAAACA-3′) and T21B14-19 (5′-ATCGGCAGATGAAGAGACTGTTTCTGCTAA-3′), and T21B14-20 (5′-ATCAGGCTTCTTCCTTGAAGTTGCCATTGCA-3′) and T21B14-21 (5′-TTGGTAACGTAACTGTAAATCTTCTCT-3′). A G-to-A mutation was found in the 3′-splice site preceding exon 8 at nucleotide 2003 (where 1 is the A in the initiator ATG).

The *mtp5-2* mutant is a sequence-indexed *Arabidopsis* T-DNA insertion mutant (GABI_351G01) isolated by the GABI-Kat facility (Rosso *et al.* 2003). We verified the position of the T-DNA insert in *mtp5-2* by using PCR amplification with the oligonucleotides MTPc2-12 (5′-TGTAGTCAAGCAAGAAGACACTTTTATTCA-3′) and MTPc2-13 (5′-TATGCTGCAGCCTACAGAAAAGCAGAAGAT-3′) and the T-DNA specific primer, LB1-GABI (5′-CTTTCTTTTTCTCCATATTGACCATCA-3′). PCR amplification of MTPc2-12 and MTPc2-13 yielded a 309 bp product from wild-type genomic DNA, whereas amplification with MTPc2-13 and LB1-GABI yielded a 250-bp product from *mtp5-2* genomic DNA. This product was sequenced, revealing that the T-DNA insertion is located in exon 7 at position 1873 in *MTP5* (where 1 is the A in the initiator ATG).

*ilr1-5* is a missense mutation the Col-0 accession (Rampey *et al.* 2006) that was backcrossed to Col-0 five times prior to crossing with *mtp5-1*. *iar3-1* is a missense mutation in the Ws accession (Davies *et al.* 1999) that was introgressed into Col-0 by three rounds of outcrossing prior to crossing with *mtp5-1*. *mtp5-1 ilr1-5* and *mtp5-1 iar3-1* double mutants were isolated from segregating F_2_ populations using PCR amplification and restriction digest analyses. The *ilr1-5* mutation was followed via amplification with 4G12-43 (5′-CAATCATCGCTTCCGCTAC-3′) and 4G12-34 (5′-CCACGCAGCTACACCGCAC-3′) and digestion with *Rsa*I, resulting in 299- and 275-bp products for *ILR1* DNA and a 574-bp product for *ilr1-5* DNA. Homozygous *mtp5-1* and *iar3-1* plants were identified by following derived cleaved amplified polymorphic sequences (Michaels and Amasino 1998; Neff *et al.* 1998). Amplification with MTPc2-1 (5′-CCTAAACATAGGACCTCTGCATTTTCAAGC-3′) and MTPc2-2 (5′-TGCATCAAACTTATATACAGTCAACATGAA-3′) yields a 219-bp product. The altered nucleotide in MTPc2-1 (underlined) creates a *Hin*DIII restriction site in the *mtp5-1* product to give 189- and 30-bp products after digestion, whereas the *MTP5* product is not cleaved by *Hin*DIII. The *iar3-1* mutation was followed by amplification with ILL4-22 (5′-CCTGTGAGTCTAAAGGATCTGCCTCTCGTG-3′) and ILL4-24 (5′-CAAATCAATTGGCATTAGGTCAAGTAAGCT-3′). ILL4-24 creates a *HinD*III site (underlined nucleotide) in the *iar3-1* PCR product resulting in 174- and 30-bp products after digestion, whereas the 200 bp *IAR3* product remains uncut.

### *MTP5* cDNA isolation

To isolate a *MTP5* cDNA, RNA was isolated with RNeasy Mini Kits (QIAGEN, Valencia, CA) from 7-d-old Col-0 seedlings grown on filter paper in 150-mm plates containing PNS at 22° in yellow-filtered light. Reverse transcriptase (RT) reactions used RETROScript (Ambion, Austin, TX) on the Col-0 RNA primed with the oligonucleotide MTPc2-4 (5′-TTGCGGCCGCACCATGATCTCTAGTATACATCC-3′). The resulting cDNA was PCR-amplified using *Pfu* Turbo DNA polymerase (Stratagene) and the oligonucleotides MTPc2-4 and MTPc2-3 (5′-TCGGATCCTCGACGAAGTTGGAACTTTAAGATC-3′). Underlined base pairs in MTPc2-3 and MTPc2-4 were altered to create a *Sal*I or *Not*I restriction site, respectively, for subsequent subcloning. The *MTP5* RT-PCR product was cloned into the pCR4Blunt-TOPO vector (Invitrogen, Carlsbad, CA) and transformed into TOP10 *Escherichia coli* (Invitrogen, Carlsbad, CA). Sequence analysis of the TOPO-MTP5 plasmid showed that the *MTP5* sequence was mis-spliced (5′ of the *mtp5-1* mutation) compared to annotation by The *Arabidopsis* Information Resource (TAIR; www.arabidopsis.org). Sequence analysis of the RT-PCR product revealed not only the cloned mis-spliced cDNA, designated *MTP5-B*, but also a second product that matched the predicted *MTP5* sequence, designated *MTP5-A*.

Using this sequence information, *MTP5-B* was excised from pCR4Blunt-TOPO with *Eco*RI and ligated into pBluescript KS (+) (Stratagene) cut with *Eco*RI to obtain pKS-MTP5premut. *MTP5-B* is oriented in the opposite orientation as LacZ in pKS-MTP5premut. Site-directed oligonucleotide-mutagenesis (Ausubel *et al.* 1999) was performed using MTPc2-10 (5′-TTTACTCCGTTGATGGAAGTGATGTGTTTTTCGG-3′) and MTPc2-11 (5′-ATCTGCTTTCACTAATGCTCTGTTCCTTATGTTCAT-3′) on pKS-MTP5premut. The underlined base pair in MTPc2-10 was altered to change the A to a G at nucleotide 226 (where 1 is the A in the initiator ATG of the cDNA), which caused an amino acid change from Asn to Ser. MTPc2-11 removed four nucleotides (ACAG) after nucleotide 563 in the cDNA, which changed the splicing pattern into that identified for *MTP5-A*. The mutagenized cDNA, pKS-MTP5, was identified by PCR amplification and restriction analyses. The *MTP5* coding sequence was amplified with T21B14-20 + MTPc2-7. *MTP5-B*, but not *MTP5-A*, contains a G at position 226, so the resulting product was digested with *Tsp*RI, which cleaves *MTP5-B* sequence but not *MTP5-A*. In addition, the PCR product resulting from amplification with T21B14-19 and MTPc2-9 was digested with *Pvu*II, because this site was destroyed when the 4 nucleotides were removed.

### *MTP5* overexpression in plants

The *MTP5* cDNA was excised from pKS-MTP5 with *Sal*I and *Not*I, restriction sites incorporated at the cDNA ends during the initial RT-PCR amplification, and was ligated into the 35S-pBARN expression vector (LeClere and Bartel 2001) cut with *Xho*I and *Not*I. The resultant pBARN-*MTP5* plasmid was electroporated (Ausubel *et al.* 1999) into *Agrobacterium* GV3101 cells (Koncz *et al.* 1992) and transformed into Col-0 and *mtp5-1 iar1-3* plants using the floral dip method (Clough and Bent 1998). Transformants were selected on PN containing 10 μg/mL Basta, and homozygous lines were selected by following Basta resistance in subsequent generations.

### RT-PCR analysis

RT-PCR analysis to identify the *mtp5-1* mutant coding sequence was conducted using RNA from leaves of a homozygous *mtp5-1 iar1-3* (N13) backcrossed plant as described previously. Sequencing the RT-PCR product with oligonucleotides used for amplification showed that the *mtp5-1* mutation shifts the splice site 1 bp 3′ of the original site. This shift causes a frameshift of the coding sequence that results in a stop codon after 33 bp.

To distinguish between *MTP5-A* and *MTP5-B*, we reverse transcribed the two transcripts were as described previously by using DNaseI-treated (Amplification Grade, Roche Applied Science, Indianapolis, IN) RNA from Col-0 7-d-old seedlings and the oligonucleotide MTPc2RT-1 (5′-TTCATCTTGTATAAATGCATGAAGAGCTT-3′). cDNAs were amplified with MTPc2RT-2 (5′-GTGTTGTATTCTACAACAGAGCTCTCTAT-3′) and MTPc2RT-1, and the resulting products were digested with *Pvu*II to give a 312-bp product for *MTP5-A* and 246- and 66-bp products for *MTP5-B*.

RT-PCR analysis also was completed to determine whether *mtp5*-2 expresses any intact *MTP5* mRNA. RNA was isolated from *mtp5-1*, *mtp5-2*, and Col-0 7-d-old seedlings as described previously and RT was performed using MTPc2-RT-4 (5′-CCATCTGAAGCAAGACACCACCAGTGGCTT-3′) and the resulting cDNA was amplified using MTPc2-RT-3 (5′-CGTTTGCTTGCACGTCATATCAGATTCCAT-3′) and MTPc2-RT-4 to yield several splicing products spanning the *mtp5* mutations. Products detected were unspliced (404 bp), fully spliced (180 bp), intron 6 present (312 bp), and intron 7 (274 bp).

### *MTP5* yeast expression

The *MTP5-A* cDNA was excised from pKS-*MTP5* with *Bam*HI and *Not*I and ligated into *Bam*HI/*Not*I-cut-pTGPD, a yeast expression vector that contains the glyceraldehyde-3-phosphate dehydrogenase constitutive promoter and the *TRP1* biosynthetic gene as a selectable marker (Chang and Lindquist 1994). The resulting plasmid, pTGPD-MTP5, was sequenced with the oligonucleotides MTPc2-3 and T21B14-19 to verify the orientation of the *MTP5* cDNA.

To test whether *MTP5* could complement yeast metal transport mutants, pTGPD-MTP5 or the empty pTGPD vector were transformed (Gietz and Schiestl 1995) into cm100 (wild type), cm102 (*zrc1*), cm103 (*cot1*), and cm104 (*zrc1 cot1*) yeast strains (Eide *et al.* 1996). Transformations were plated on selective media (Ausubel *et al.* 1999) lacking Trp for cm100, lacking Trp and His for cm102, lacking Trp and Ura for cm103, and lacking Trp, His, and Ura for cm104. For metal response analysis, positive transformants were streaked on YPD (Ausubel *et al.* 1999) plates with no metals or containing ZnSO_4_ or CoCl_2_ and incubated at 30° for 3 d.

## Results

### Isolation of a mutant defective in the cation diffusion facilitator MTP5

The *iar1* mutant displays reduced root elongation inhibition on several IAA-amino acid conjugates, including IAA-Ala (Lasswell *et al.* 2000). To identify genes acting with *IAR1* to control conjugate sensitivity, we conducted a genetic modifier screen to isolate extragenic mutations that suppressed the *iar1* mutant phenotype. We screened approximately 48,000 progeny of mutagenized *iar1* seeds for restored sensitivity to 40 μM IAA-Ala and isolated 61 putative suppressors, of which seven set seeds and retained wild-type IAA-Ala sensitivity in the next generation. One of these suppressors was selected for further analysis.

**Figure 1:**
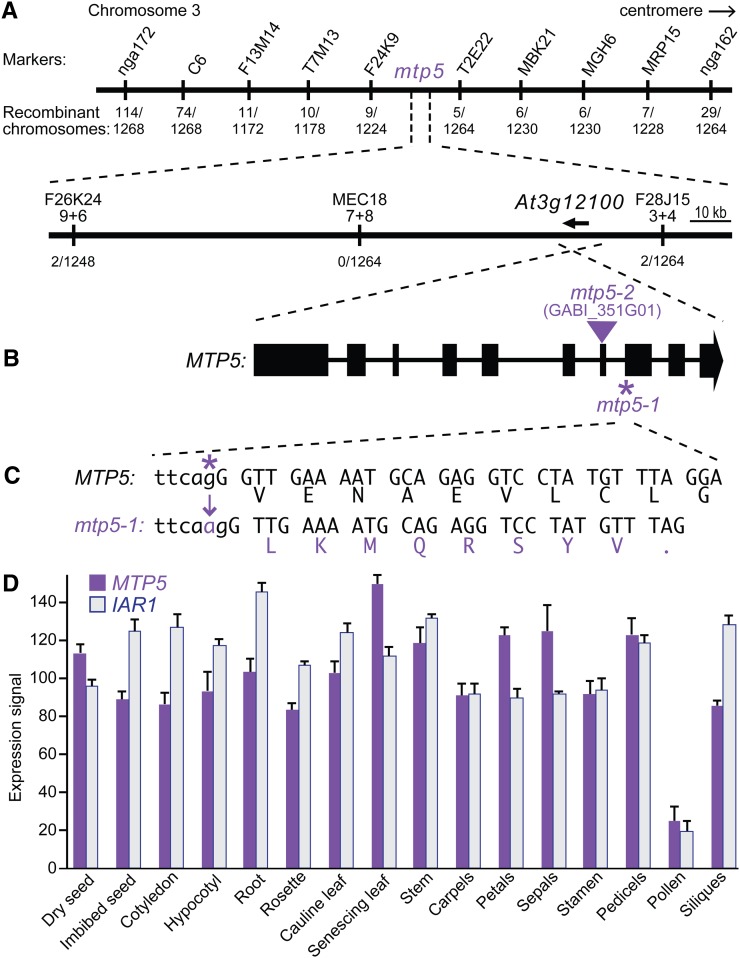
Positional cloning of the gene defective in *mtp5-1*. (A) Recombination mapping of *mtp5-1*. The lesion suppressing *iar1* IAA-Ala resistance was mapped to chromosome 3 between markers F26K24-9+6 and F28J15-3+4 (Table 1), an interval that includes the *MTP5* (*At3g12100*) gene. (B) *MTP5* contains 10 exons (boxes) separated by 9 introns (lines). The location of the *mtp5-2* T-DNA insertion is indicated by the triangle. (C) The G-to-A mutation in *mtp5-1* is located at position 2003 (where 1 is the A in the initiator ATG) and alters the 3′ splice site of the 7th intron (lower-case letters are intronic bases; capital letters are exonic base pairs). RT-PCR analysis revealed that the 3′ splice site in the *mtp5-1* mutant occurs 1 bp 3′ of the wild-type site, resulting in a frameshift and a premature termination codon. (D) Relative expression levels of *MTP5* and *IAR1* mRNAs in selected Arabidopsis tissues. Compiled microarray data were retrieved from the Arabidopsis eFP Browser (http://bar.utoronto.ca/efp/) in October 2012. Tissues queried were dry and imbibed (24 hr) seeds; cotyledons and hypocotyls from 7-d-old seedlings; roots from 17-d-old seedlings; vegetative rosettes from 14-d-old plants; cauline leaves from 21-d-old plants; senescing leaves from 35-d-old plants; stems from 21-d-old plants; carpels, petals, sepals, stamen, and pedicels from stage 15 flowers; mature pollen; and stage 3 siliques. Error bars show SD of mean expression signals scaled to a target intensity value of 100 for each gene.

We used PCR-based markers to map the suppressor mutation to the top of chromosome three between nga172 and nga162 (Figure 1A, Bell and Ecker 1994). After identifying new molecular markers within this interval (Table 1), we narrowed the mapping region to ~160 kb between the markers F26K24-9+6 and F28J15-3+4 (Figure 1A). Using a candidate gene sequencing approach, we identified a mutation in the *iar1* suppressor mutant in the gene encoding a putative cation diffusion facilitator/metal transport protein, MTP5 (At3g12100; Figure 1B). The *mtp5-1* mutation is a G-to-A change at nucleotide 2003 (where 1 is the A in the initiator ATG) in the 3′ splice site preceding exon 8 (Figure 1C). We sequenced *MTP5* in our other *iar1* suppressors and did not recover any additional *mtp5* alleles.

**Figure 2:**
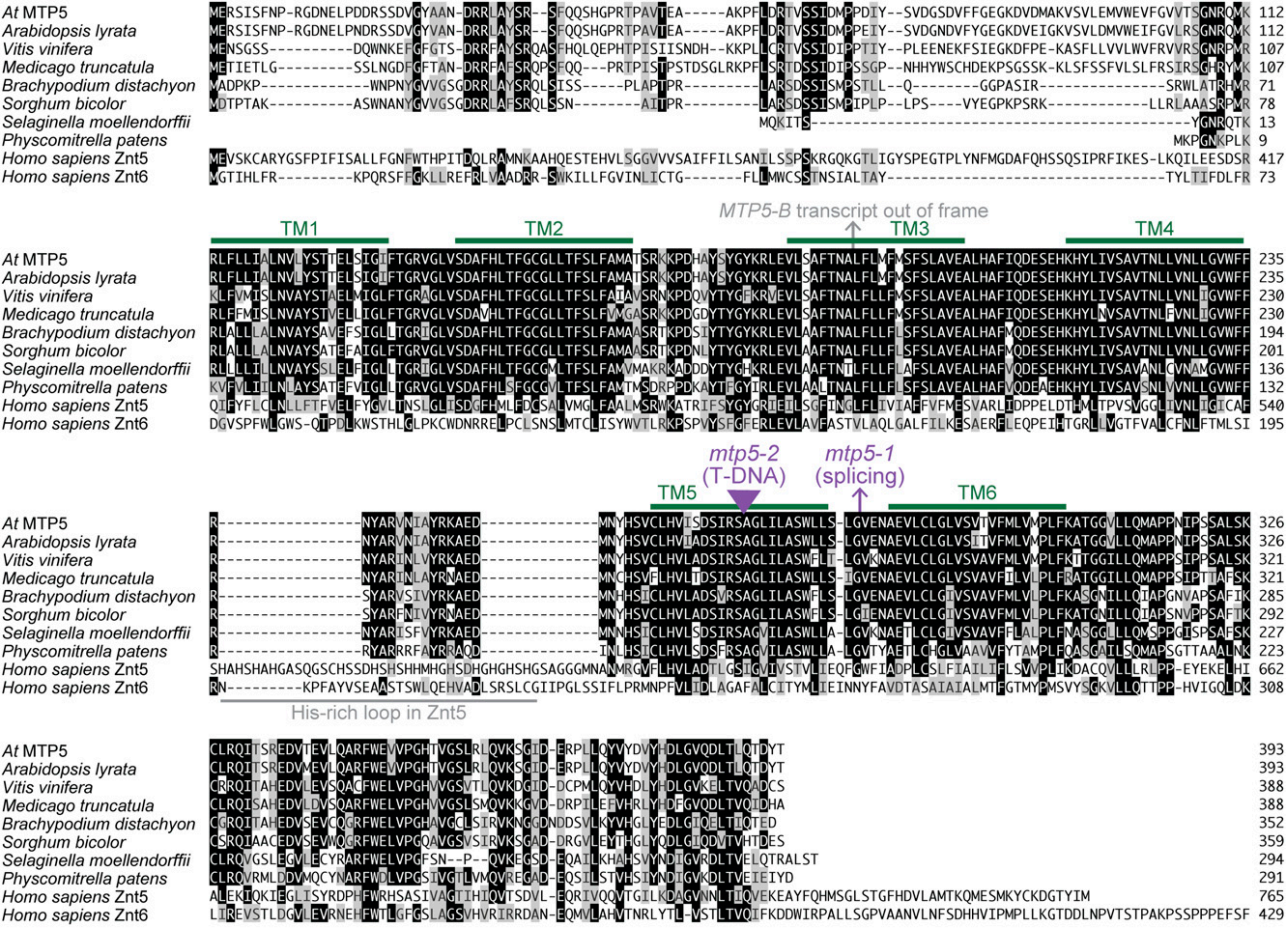
Alignment of *Arabidopsis* MTP5 and related proteins. *Arabidopsis thaliana* MTP5 (At3g12100; AAT44130.1) was aligned with likely orthologs from other plants (*Arabidopsis lyrata* XP_002884882.1, *Vitis vinifera* XP_002279787.1, *Medicago truncatula* XP_003625268.1, *Sorghum bicolor* XP_002453105.1, *Brachypodium distachyon* XP_003570736.1, *Selaginella moellendorffii* XP_002982633.1, *Physcomitrella patens* XP_001755969.1) and the human ZnT5 (*Homo sapiens* NP_075053.2; residues 296-765 of 765 residues) and ZnT6 (*Homo sapiens* NP_001180442; residues 1-429 of 501 residues) zinc transporters using the MegAlign program (DNAStar) using the Clustal W method. Residues identical in at least five sequences are shaded in black boxes; chemically similar residues in at least five sequences are shaded in gray boxes. Potential transmembrane (TM) domains in *Arabidopsis thaliana* MTP5 predicted with Aramemnon (Schwacke *et al.* 2003) are marked with green lines; the His-rich loop found in Znt5 but not MTP5 orthologs is marked by a gray line. The position of the alternative splicing events that would lead to an out-of-frame sequence followed by premature termination codons caused by the *mtp5-1* mutation or detected in wild-type mRNA (the *MTP5-B* transcript) are indicated by arrows. The position of the *mtp5-2* T-DNA disruption is indicated with a triangle.

To determine the molecular consequences of the *mtp5-1* mutation, we isolated RNA from *mtp5-1* plants and reverse-transcribed and PCR-amplified the *mtp5-1* locus. Sequencing the RT-PCR product revealed that the *mtp5-1* coding sequence is spliced 1 nucleotide after wild type, creating a frameshift and a premature stop codon nine codons after the splicing mutation (Figure 1C). The mtp5-1 protein, if stable, would thus only encode five of the six conserved transmembrane domains (Figure 2), presumably precluding transport function.

To determine whether *MTP5* was expressed in similar tissues as *IAR1*, we examined publicly available microarray datasets (Winter *et al.* 2007). We found that *MTP5*, like *IAR1*, was widely present at similar levels across many tissues (Figure 1D), consistent with the possibility that the MTP5 and IAR1 proteins might influence similar functions.

### *mtp5* restores IAA-conjugate sensitivity to *iar1*

**Figure 3:**
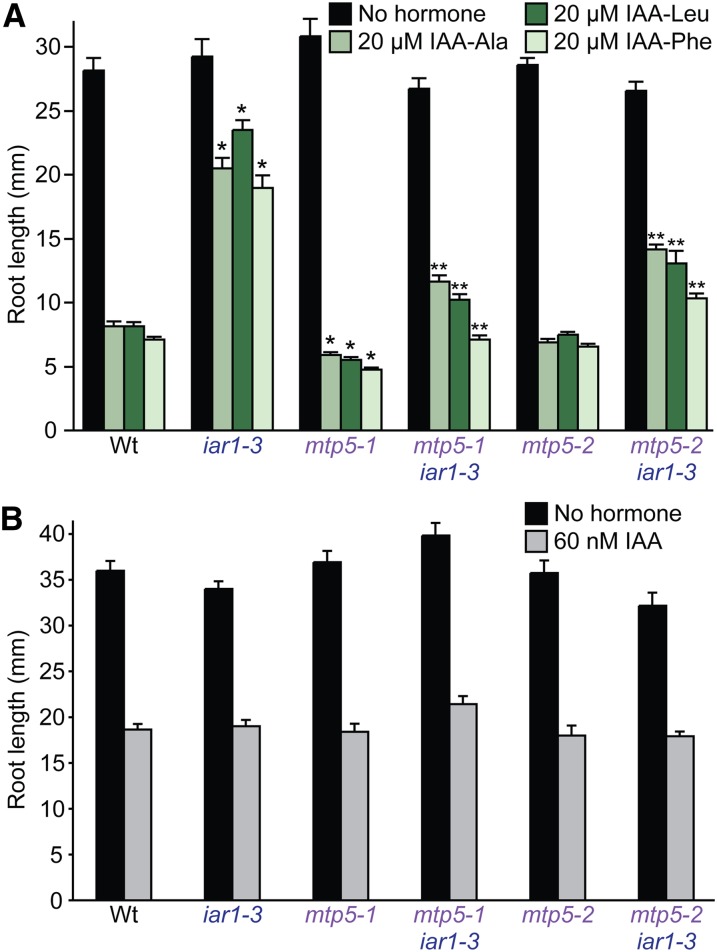
(A) *mtp5* mutations restore IAA-conjugate sensitivity to *iar1* roots. Col-0 (Wt), *iar1-3*, *mtp5-1*, *mtp5-2*, *mtp5-1 iar1-3*, and *mtp5-2 iar1-3* seedlings were grown on unsupplemented medium or medium containing 20 µM IAA-Ala, IAA-Leu, or IAA-Phe. (B) Like *iar1*, *mtp5*, and *mtp5 iar1* mutants respond normally to IAA. Seedlings listed in (A) were grown on unsupplemented medium or medium containing 60 nM IAA. Seedlings were grown in constant light under yellow filters for 8 (A) or 9 (B) d at 22°. Error bars indicate standard errors of the mean root lengths (n = 12). Single asterisks indicate single mutant root lengths significantly different from Wt; double asterisks indicate double mutant root lengths significantly different from *iar1-3* (two-tailed *t*-tests; *P* < 0.001).

*iar1* mutants are resistant to a variety of IAA-amino acid conjugates, including IAA-Ala, IAA-Leu, and IAA-Phe (Lasswell *et al.* 2000). We found that the *mtp5-1* mutation was recessive (data not shown) and restored *iar1* root sensitivity not only to IAA-Ala, which we used to isolate the mutant, but also to IAA-Leu and IAA-Phe (Figure 3A). This suppression did not result from generalized auxin resistance because *iar1-3 mtp5-1* seedling roots retained wild-type sensitivity to the inhibitory effects of IAA on root elongation (Figure 3B). Beyond restored IAA-amino acid conjugate sensitivity, *iar1-3 mtp5-1* seedlings did not exhibit obvious morphological abnormalities and displayed wild-type numbers of lateral roots and normal hypocotyl lengths (data not shown).

Interestingly, when the *mtp5-1* mutation was removed from the *iar1* mutant background, *mtp5-1 IAR1* seedlings were slightly more sensitive to root elongation inhibition by IAA conjugates than wild-type seedlings (Figure 3A). The single mutant was not more sensitive to free IAA (Figure 3B), however, again indicating that MTP5 specifically dampens responsiveness to IAA-amino acid conjugates.

### Alternative splicing of *MTP5* transcripts

**Figure 4:**
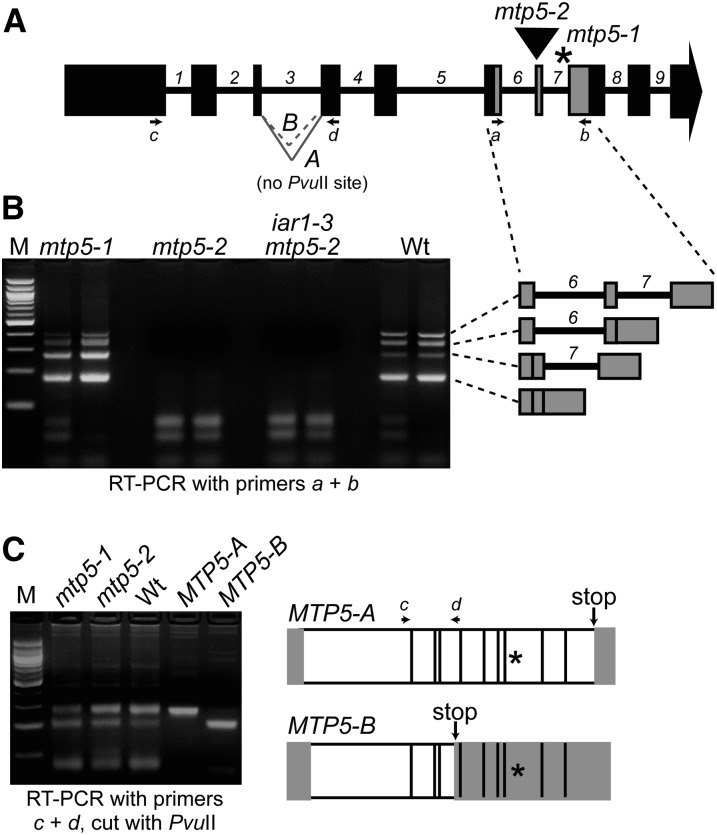
Alternative splicing of *MTP5* transcripts. (A) Model of *MTP5* gene showing exons (boxes), introns (numbered), positions of *mtp5* mutations (asterisk, *mtp5-1*; triangle, *mtp5-2*), primers used for RT-PCR amplification (arrows a-d), and the A and B *MTP5* splice variants of intron 3. (B) *mtp5-2* seedlings lack intact *MTP5* transcript, and *mtp5-1* and wild-type seedlings inefficiently splice *MTP5* introns 6 and 7. *MTP5* transcripts were detected via RT-PCR amplification using gene-specific primers (A and B) flanking both *mtp5* mutations with RNA isolated from 7-d-old *mtp5-1*, *mtp5-2*, *iar1-3 mtp5-2*, and Col-0 (Wt) seedlings. Adjacent lanes show PCR-amplification products of cDNA from two independent reverse transcription reactions using the same RNA. The identities of the four amplicons from the RT-PCR analysis using primers a and b were determined by sequencing and are shown to the right in gray. (C) *mtp5-1* and wild-type seedlings alternatively splice *MTP5* intron 3. Using gene-specific primers c and d (A), *MTP5* transcripts were amplified from cDNA reverse-transcribed from Col-0 (Wt), *mtp5-1*, and *mtp5-2* RNA. RT-PCR analysis revealed two transcripts in wild type and the two mutant alleles, designated *MTP5-A* and *MTP5-B*. Digesting the PCR products with *Pvu*II distinguished *MTP5-A*, which lacks a *Pvu*II site, from *MTP5-B*, which includes a *Pvu*II site (agarose gel image in C). Cloned *MTP5-A* and *MTP5-B* cDNAs were included as positive controls. The transcripts were differently spliced at the 3′ end of the third intron, resulting in a premature stop codon (arrows) in *MTP5-B*. Asterisks mark the position of the *mtp5-1* mutation. White boxes indicate the open reading frame of the resultant transcripts, and gray boxes show predicted untranslated regions.

We obtained a second *mtp5* allele, designated *mtp5-2*, from the GABI-Kat collection of sequence-indexed T-DNA insertion mutants (Rosso *et al.* 2003). Sequencing the insertion site revealed that the T-DNA was inserted in the seventh *MTP5* exon (Figure 4A) near the site of the *mtp5-1* lesion (Figure 2), and RT-PCR analysis confirmed that the *mtp5-2* allele lacked intact *MTP5* RNA (Figure 4B).

In the course of analyzing *mtp5-2* RNA, we found that the *MTP5* introns 6 and 7 were inefficiently spliced in both wild-type and *mtp5-1* seedlings. In addition to fully spliced product, we detected partially processed *MTP5* mRNA (Figure 4B). Interestingly, the partially spliced transcript that retained the 7th intron appeared to accumulate more in *mtp5-1* than in wild type (Figure 4B), providing evidence that the *mtp5-1* splice site mutation at the 3′ end of intron 7 (Figure 1C) affects not only the position, but also the efficiency of *MTP5* transcript splicing.

In addition to the inefficient splicing of *MTP5* introns 6 and 7, we found that intron 3 was alternatively spliced in both wild type and *mtp5* mutants. We detected two fully spliced *MTP5* transcripts, *MTP5-A* and *MTP5-B*, in 7-d-old seedlings; *MTP5-A* was more abundant than *MTP5-B* (Figure 4C). These transcripts used different 3′ splice sites of the third intron (Figure 4C); the *MTP5-B* transcript would truncate the encoded protein in the third predicted transmembrane domain (Figure 2). Because the stop codon in *MTP5-B* is at a position prior to the *mtp5-1* mutation in intron 7, we concluded that it is the *MTP5-A* transcript that is defective in *mtp5-1*.

### Characterization of a second *mtp5* allele

**Figure 5:**
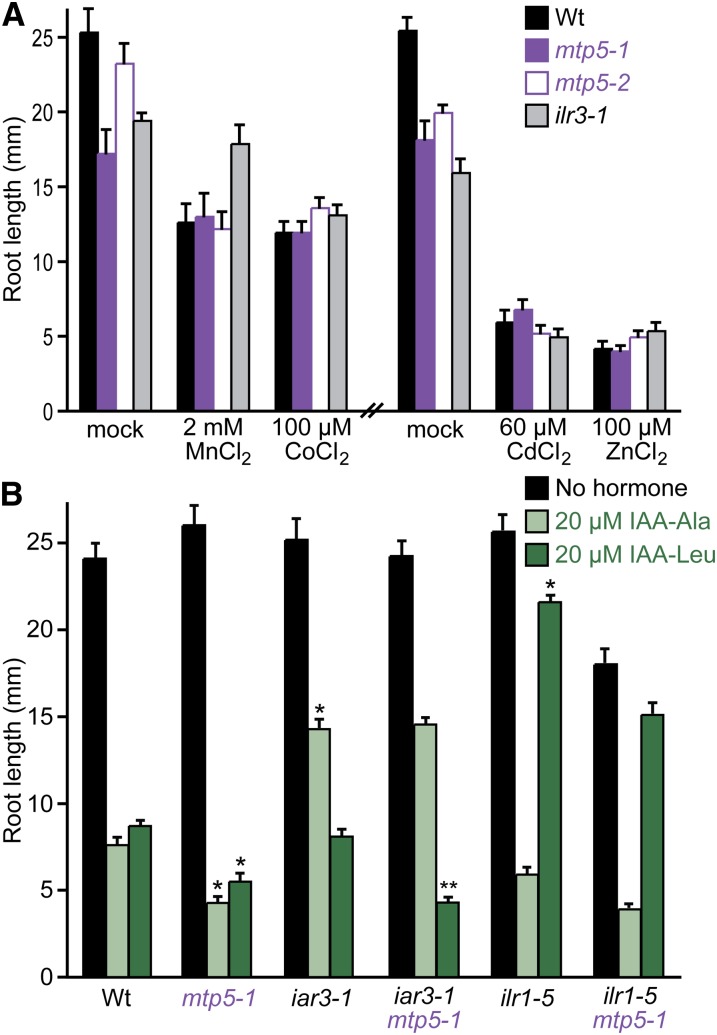
*mtp5* mutations do not alter metal sensitivity or restore IAA-amino acid sensitivity to IAA-conjugate hydrolase mutants. (A) *mtp5* mutant roots respond normally to inhibitory concentrations of various metals. Col-0 (Wt), *mtp5-1*, *mtp5-2*, and *ilr3-1* seedlings were grown on unsupplemented medium or medium containing 2 mM MnCl_2_, 100 µM CoCl_2_, 60 µM CdCl_2_, or 100 µM ZnSO_4_ in constant light under yellow filters for 8 d at 22°. Error bars indicate standard errors of the mean root lengths (n ≥ 9). (B) *mtp5-1* does not suppress the IAA-conjugate resistance of mutants defective in IAA-amino acid hydrolases. Col-0 (Wt), *iar3-1*, *ilr1-5*, *mtp5-1*, *iar3-1 mtp5-1*, and *mtp5-1 ilr1-5* seedlings were grown on unsupplemented medium or medium containing 20 µM IAA-Ala or IAA-Leu in constant light under yellow filters for 8 d at 22°. Error bars indicate SE of the mean root lengths (n ≥ 9). Single asterisks indicate single mutant root lengths significantly different from Wt; double asterisks indicate *iar3-1 mtp5-1* double mutant root lengths significantly different from *iar1-3* (two-tailed *t*-tests; *P* < 0.001). Significance was not calculated for *ilr1-5 mtp5-1* differences because the double mutant roots were shorter than the single mutant roots on unsupplemented medium.

To confirm that the mutation that we identified in *mtp5-1* caused the observed suppression of the IAA-conjugate resistance of *iar1*, we compared phenotypes of our original *mtp5-1* splicing allele and the *mtp5-2* T-DNA insertion allele. We crossed *mtp5-2* to the *iar1-3* mutant to determine whether this allele also restored IAA-conjugate sensitivity to *iar1*. Indeed, *iar1-3 mtp5-2* seedlings were significantly more sensitive to IAA-amino acid conjugates than *iar1-3* (Figure 3A), confirming that the *mtp5-1* splice-site mutation is a loss-of-function allele and that reducing MTP5 function can compensate for the lack of IAR1 activity in an *iar1* mutant. Unlike *mtp5-1* (Figure 3A and 5B), *mtp5-2* in an otherwise wild-type background was not significantly hypersensitive to IAA conjugates (Figure 3A).

Because MTP5 is a metal transporter homolog, we examined *mtp5* sensitivity to various metals in root growth inhibition assays. Unlike the *ilr3-1* mutant, which is specifically resistant to Mn^2+^ (Rampey *et al.* 2006), both *mtp5-1* and *mtp5-2* (in the wild-type *IAR1* background) displayed responses similar to wild type to all of the metals tested, including Mn^2+^, Zn^2+^, Co^2+^, and Cd^2+^ (Figure 5A). Moreover, we found that driving expression of the *MTP5-A* cDNA in wild-type Col-0 seedlings behind the strong CaMV 35S promoter did not alter seedling root lengths on medium supplemented with one of several metals, including Mn^2+^, Zn^2+^, Co^2+^, and Cd^2+^ (data not shown).

### *mtp5-1* does not suppress other IAA-conjugate sensitivity mutants

To determine whether *mtp5-1* could suppress the IAA-conjugate resistance of mutants in addition to *iar1*, we constructed double mutants of *mtp5-1* with other IAA-conjugate resistant mutants, including the IAA-amino acid conjugate amidohydrolase mutants *iar3-1* (Davies *et al.* 1999) and *ilr1-5* (Rampey *et al.* 2006), which are resistant to the inhibitory effects of IAA conjugates that are substrates of the mutated hydrolases. For example, *iar3* is resistant to IAA-Ala and not IAA-Leu and IAR3 cleaves IAA-Ala more efficiently than IAA-Leu *in vitro* (Davies *et al.* 1999), whereas *ilr1* is resistant to IAA-Leu and not IAA-Ala and ILR1 cleaves IAA-Leu more efficiently than IAA-Ala *in vitro* (Bartel and Fink 1995). We examined root lengths of each double mutant after growth on IAA-amino acid conjugates. We found that *mtp5-1* failed to restore *ilr1-5* sensitivity to IAA-Leu or *iar3-1* sensitivity to IAA-Ala, and that the heightened sensitivity of *mtp5-1* to IAA-Leu was retained in the *iar3-1 mtp5-1* double mutant (Figure 5B). This lack of suppression of the amidohydrolase mutants establishes that *mtp5-1* is not a general suppressor of all conjugate response mutants. Moreover, the epistatic relationship of these mutants demonstrates that the increased IAA-conjugate sensitivity of the *mtp5-1* mutant requires intact conjugate hydrolases with specificity for the indicated IAA-amino acid.

## Discussion

### Plant cation diffusion facilitator (CDF) transporters

We isolated an extragenic suppressor of the IAA-conjugate response mutant *iar1* and found that the defective gene encodes MTP5, a previously uncharacterized member of the CDF transporter family found in bacteria, yeast, animals, and plants. Plant CDF proteins usually have six predicted transmembrane domains (Figure 2) flanked by an N-terminal signature sequence and a C-terminal cation efflux domain (Mäser *et al.* 2001). There are 12 members of this family in *Arabidopsis*. Phylogenetic analysis and substrate specificity of characterized members separates the CDF family into three groups: Mn-CDF, Fe/Zn-CDF, and Zn-CDF (Montanini *et al.* 2007; Gustin *et al.* 2011). MTP5 is in the Zn-CDF family; all biochemically-characterized members of this subfamily transport at least zinc (Montanini *et al.* 2007).

Although none are functionally characterized, MTP5 has apparent orthologs in a variety of plants, including *Medicago*, *Brachypodium*, *Sorghum*, *Selaginella*, and *Physcomitrella* (Figure 2), suggesting an early emergence of MTP5 during the evolution of land plants. These proteins are between 63 and 73% identical to *A. thaliana* and *A. lyrata* MTP5, which are 96% identical to each other. Less similarity is present in the region N-terminal to the first predicted transmembrane domain, which is missing in the MTP5 orthologs from *Selaginella* and *Physcomitrella*. The plant MTP5 homologs lack the His-rich loop found between transmembrane domains four and five in some zinc transporters, such as human ZnT5 (Figure 2) and *Arabidopsis* MTP1 and MTP3 (Kawachi *et al.* 2012).

Of the 12 *Arabidopsis* MTP (CDF) proteins, six are in the Zn-CDF subfamily (Montanini *et al.* 2007; Gustin *et al.* 2011). This subfamily includes the first characterized plant MTP protein, *Arabidopsis* ZAT (Van Der Zaal *et al.* 1999), now designated MTP1 (Mäser *et al.* 2001). MTP1 transports Zn^2+^ when heterologously expressed in *E. coli* proteoliposomes (Bloß *et al.* 2002), *mtp1* mutant roots accumulate less Zn^2+^ in vacuolar-like organelles (Kawachi *et al.* 2009), and transgenic plants overexpressing *MTP1* hyperaccumulate Zn^2+^ and have decreased Zn^2+^ sensitivity (Van Der Zaal *et al.* 1999). In contrast, we found no alterations in Zn^2+^ sensitivity when MTP5 was mutated or overexpressed.

Characterized AtMTP1 homologs in other plant species all are proposed to sequester metals in intracellular compartments or catalyze metal efflux from cells. For example, the *Thalspi caerulescens* (Tc) *ZTP1* gene is induced when soil is enriched with Zn^2+^, Cd^2+^, or Pb^2+^ (Assuncao *et al.* 2001). Heterologous expression of an AtMTP1 homolog from the tropical legume *Stylosanthes hamata*, ShMTP1, confers Mn^2+^ tolerance to yeast and *Arabidopsis* (Delhaize *et al.* 2003). ShMTP1-GFP fusions localize to plant tonoplasts and the yeast ER (Delhaize *et al.* 2003). Similarly, heterologous expression of PtdMTP1 from poplar (*Populus trichocarpa x P. deltoids*) confers Zn^2+^ resistance to yeast and *Arabidopsis*, and PtdMTP1-GFP fusions are vacuolar in both yeast and *Arabidopsis* (Blaudez *et al.* 2003). Interestingly, the *Thalspi goesingense TgMTP1* gene gives rise to three transcript variants, *TgMTP1a*, *TgMTP1b*, and *TgMTP1c* (Kim *et al.* 2004). Expression of *TgMTP1a* in yeast confers resistance to Cd^2+^, Co^2+^, and Zn^2+^, whereas *TgMTP1b* expression confers resistance to Ni^2+^ (Persans *et al.* 2001). TgMTP1b localizes to the plasma membrane of *Arabidopsis* leaf protoplasts and may efflux Zn^2+^ from cells (Kim *et al.* 2004). Although we also found evidence for alternative splicing of *Arabidopsis MTP5* (Figure 4), only one of these splice products (*MTP5-A*) is likely to encode an intact transporter.

Several MTP1 proteins have been tested for complementation of the yeast mutant *cot1 zrc1*, which is defective in vacuolar metal efflux (MacDiarmid *et al.* 2000). The three TgMTP1 isoforms, TcZTP1, *Thaspi arvense* MTP1, *Thaspi montanum var. fendleri* MTP1, *Arabidopsis lyrata* MTP1, AtMTP1, and PtdMTP1 all confer Zn^2+^ resistance when expressed in yeast (Blaudez *et al.* 2003; Kim *et al.* 2004). However, the authors of a previous study found that AtMTP1 did not complement the *cot1 zrc1* mutant (Bloß *et al.* 2002). Expression of TgMTPb truncations containing the N-terminus, C-terminus, the putative metal-binding His-rich domain, or various combinations of these domains do not complement the *cot1 zrc1* mutant, suggesting that the Zn^2+^ resistance that accompanies *TgMTPb* overexpression is related to transport and not due to binding excess Zn^2+^ by the His-rich domain. We transformed the *MTP5* cDNA driven by the glyceraldehyde-3-phosphate dehydrogenase constitutive promoter into wild-type and *cot1* and *zrc1* single and double mutant yeast lines but did not observe changes in relative growth in the presence of Zn^2+^ or Co^2+^ (data not shown). It is possible that *Arabidopsis* MTP5 does not function in yeast membranes. Alternatively, because the localization and identity of metals potentially transported by MTP5 are not known, this lack of complementation may be due to differences in location or metal specificity among MTP5 and the yeast Cot1p and Zrc1p proteins.

### An *Arabidopsis* cation diffusion facilitator protein mutant

Beyond the suppression of the IAA-conjugate resistance of *iar1*, *mtp5-1*, and *mtp5-2* plants resembled wild type as seedlings and adults (Figures 3 and 5 and data not shown). The *mtp5* mutants resembled wild type on media containing metals under all conditions tested (Figure 5A). In contrast, *mtp1* mutants have increased Zn^2+^ sensitivity (Kobae *et al.* 2004; Kawachi *et al.* 2009), and *mtp11* mutants (defective in a member of the Mn-CDF family) are more sensitive to Mn^2+^ (Delhaize *et al.* 2007; Peiter *et al.* 2007). RNAi lines silencing *MTP1* or *MTP3* also show Zn^2+^ hypersensitivity (Desbrosses-Fonrouge *et al.* 2005; Arrivault *et al.* 2006). It is possible that other transporters partially compensate for any loss of transport activity in the *mtp5* mutants. In this case, the *mtp5* mutation may modify subcellular metal concentration(s) adequately to compensate for the presumed transport defect in *iar1* seedlings, but not alter levels enough to cause toxic metal accumulation that would increase sensitivity to exogenous metals.

### A working model for *mtp5* suppression of *iar1*

In eukaryotes, Zn^2+^ transport into or out of the cytosol occurs through the opposing action of two protein families, the ZIPs and CDFs (Kambe *et al.* 2004, 2006). ZIP proteins transport Zn^2+^ from outside cells and from intracellular compartments into the cytosol. In an opposing manner, CDFs transport Zn^2+^ out of the cytosol and into extracellular space or intracellular compartments. Homology thus suggests that the MTP5 CDF protein effluxes metals out of cells or into a subcellular compartment, whereas the IAR1 ZIP protein is predicted to move metals into cells or out of a subcellular compartment.

**Figure 6:**
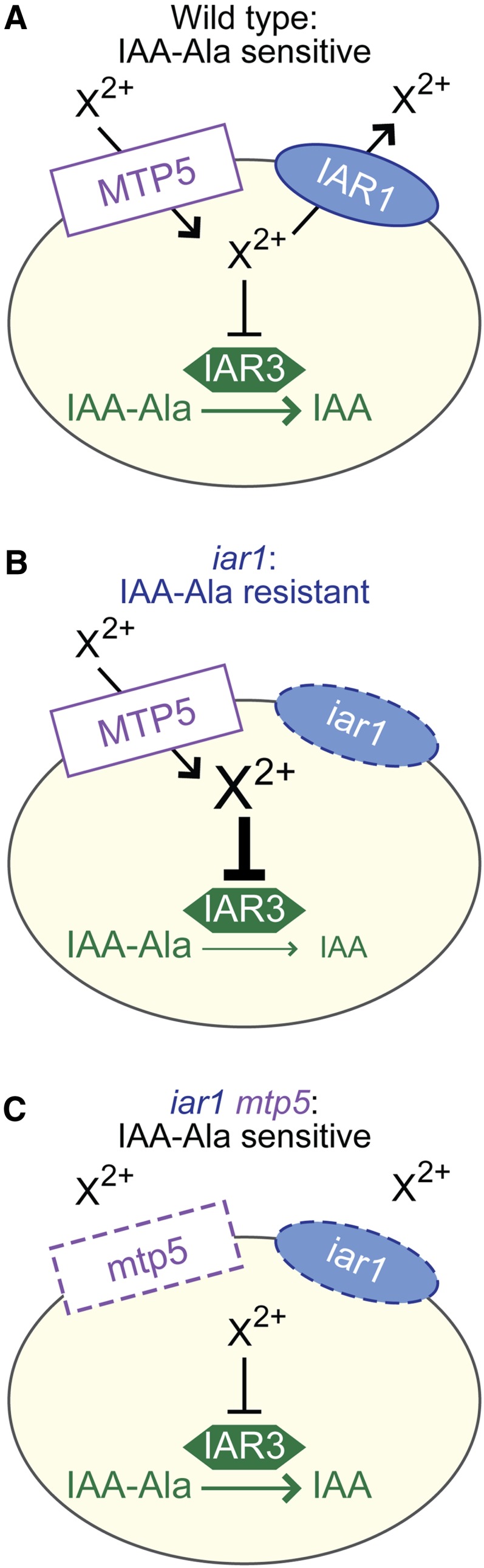
A working model for MTP5 and IAR1 function in IAA-conjugate responses in wild type (A), an *iar1* mutant (B), and an *iar1 mtp5* double mutant (C). The presence of IAA-amino acid hydrolases in the ER, the localization and function of MTP5 and IAR1 orthologs, and the *iar1* and *mtp5* mutant phenotypes (see text for details) suggest that MTP5 and IAR1 may transport Zn^2+^ or other metal(s) that inhibit hydrolase function into and out of, respectively, the ER. Validation or rejection of this model awaits localization of the MTP5 and IAR1 transporters and determination of their substrate specificities.

The mutant phenotypes and predicted protein functions suggest that MTP5 and IAR1 transport metals in an antagonistic fashion to regulate metal homeostasis, which in turn influences IAA-amino acid conjugate hydrolysis by compartmentalized enzymes (Figure 6). Based on the membrane topology of IAR1 homologs, one possibility is that IAR1 transports metals inhibitory to IAA-amino acid hydrolase function out of the compartment in which the hydrolases reside (Figure 6A). This compartment is likely to be the ER, because the yeast IAR1 ortholog Yke4p is ER-localized (Kumánovics *et al.* 2006), the hydrolases contain predicted N-terminal signal sequences and C-terminal ER retention signals (Bartel and Fink 1995; Davies *et al.* 1999), and both IAR3 and ILR1 have been localized to the ER in proteomics experiments (Dunkley *et al.* 2006). Metals impact hydrolase activity; for example, the *in vitro* activities of the IAA-conjugate hydrolases are enhanced by Mn^2+^, and ILR1 activity is inhibited by Zn^2+^ (LeClere *et al.* 2002). Moreover, the crystal structure of the *Arabidopsis* ILR1-like homolog ILL2 reveals a di-metal binding site in the hydrolase active site that is typical of the M20 metallopeptidases family (Bitto *et al.* 2009). The IAA-conjugate resistance of *iar1* may result from an aberrant accumulation of inhibitory metals in the hydrolase compartment, which decreases hydrolysis of and therefore sensitivity to IAA-amino acid conjugates (Figure 6B). If MTP5 transports inhibitory metals into the hydrolase-containing compartment, an *mtp5 iar1* mutant would no longer be IAA-conjugate resistant because inhibitory metals would not accumulate, thus restoring wild-type levels of IAA-conjugate hydrolysis (Figure 6C). This model is consistent with our observation that double mutants defective in both *MTP5* and the *ILR1* or *IAR3* hydrolase genes remain resistant to IAA-Leu or IAA-Ala (Figure 5B), respectively, indicating that MTP5 functions upstream of the hydrolases.

This compensatory model is consistent with the function of yeast transporters similar to IAR1 and MTP5. When yeast are grown in low zinc medium, the IAR1 ortholog Yke4p moves zinc from the secretory pathway into the cytosol (Kumánovics *et al.* 2006). Intriguingly, loss of Yke4 can compensate for the loss of the Msc2p (Kumánovics *et al.* 2006), a CDF protein in the MTP5 subfamily that transports zinc into the ER (Li and Kaplan 2001; Ellis *et al.* 2005).

Whereas the model depicted in Figure 6 suggests that IAR1 and MTP5 occupy the same membrane, it is also possible that either or both MTP5 and IAR1 are located in a compartment that does not contain the hydrolases. When metal homeostasis is disrupted within this compartment, it may indirectly affect the metal environment of the ER, thus indirectly affecting IAA-conjugate hydrolysis. Distinguishing between these possibilities will be aided by characterization of the subcellular localizations and metal transport specificities of the IAR1 and MTP5 proteins. Regardless of the subcellular localization of IAR1 and MTP5, however, it is clear that genetic studies of IAA-amino acid conjugate responsiveness are exquisitely sensitive to uncovering factors contributing to metal homeostasis. As this screen has not reached saturation, it is possible that continued studies will reveal additional components necessary to control the metal environment in which the hydrolases function.

Interestingly, CDF metal transporters may directly interact with a component from a signaling pathway to modify the binding partner’s activity (Jirakulaporn and Muslin 2004). The C-terminal intracellular portions of mammalian Znt1 and worm CDF-1 bind the amino-terminal regulatory region of Raf-1, a protein kinase that regulates cell proliferation and differentiation (Jirakulaporn and Muslin 2004). Further, Raf-1 activity is dependent on Znt1 function, and *in vitro* binding does not occur upon addition of Zn^2+^ (Jirakulaporn and Muslin 2004). Some CDF transporters also form heterodimers, including yeast Msc2p and Zrg17p (Ellis *et al.* 2005) and the corresponding mammalian homologs ZnT5 and ZnT6 (Ellis *et al.* 2005; Fukunaka *et al.* 2009). Identification of *Arabidopsis* MTP5 binding partners may further elucidate the relationship between metal homeostasis and IAA-conjugate sensitivity.
